# A proximal type epithelioid sarcoma of the vulva with multiple distant metastases: A case report and review of the literature

**DOI:** 10.1016/j.gore.2021.100835

**Published:** 2021-07-20

**Authors:** Hyewon Chung, Tae-Kyu Jang, Sun Young Kwon, Jinkyeong Ha, So-Jin Shin

**Affiliations:** a Department of Obstetrics and Gynecology, Keimyung University School of Medicine, Daegu, South Korea; b Department of Pathology, Keimyung University School of Medicine, Daegu, South Korea

**Keywords:** **E**pithelioid sarcoma, Proximal type, Vulva, Metastasis

## Abstract

Epithelioid sarcomas (ESs) of the vulva are extremely rare soft tissue tumors characterized by an aggressive clinical course and a poor prognosis. The proximal type of ES occurs in the trunk and external genital area and has higher recurrence and distant metastasis rates than the distal type, which is found in the upper and lower extremities. We describe a case of a vulvar ES in a 24-year-old patient who was referred from the department of plastic surgery with protruding mushroom-like lesions in multiple areas, including the lower abdomen, whole vulva, anus, and both inguinal lesions. A biopsy of the lesions confirmed a proximal-type ES. Computed tomography and magnetic resonance imaging revealed multiple metastatic lesions in several regions, including the perineum, vagina, and inguinal regions; nodal metastases in the left external iliac and right inguinal region; and distant metastases in the lungs, pleura of the left lung, bones, and soft tissue. The patient underwent active palliative radiotherapy, followed by chemotherapy, and showed a partial response to treatment. Nineteen months after the initial diagnosis, the patient expired due to cancer progression and pneumothorax.

## Highlights

• Epithelioid sarcomas (ESs) are rare and aggressive malignancy of a poor prognosis.
• Radiotherapy may be an alternative treatment to decrease the size of the lesion.
• Targeted therapy with pazopanib may be a treatment option for soft tissue sarcomas.

## 1 Introduction

Epithelioid sarcomas (ESs) are malignant soft tissue tumors, which are classified as distal or proximal types based on their occurrence in the upper and lower extremities or in the trunk and pubic regions, respectively. ESs are a very rare kind of tumor, which is characterized by aggressive biological behavior and a poor prognosis (Patrizi et al., 2013). Their natural history typically involves local recurrence and usually distant metastasis. Due to their rarity, ESs are frequently misdiagnosed, and there are no well-established guidelines on optimal treatment. Proximal types of ES are reported to have a poorer prognosis than distal types due to higher local recurrence and distant metastasis rates. Proximal-type vulvar ESs are particularly rare and occur most frequently in the labia majora of premenopausal women (Han et al., 2016). Here, we describe a case of a proximal-type vulvar ES accompanied by multiple distant metastases in a 24-year-old patient with a noteworthy overall survival.

## 2 Case report

**Fig. 1:**
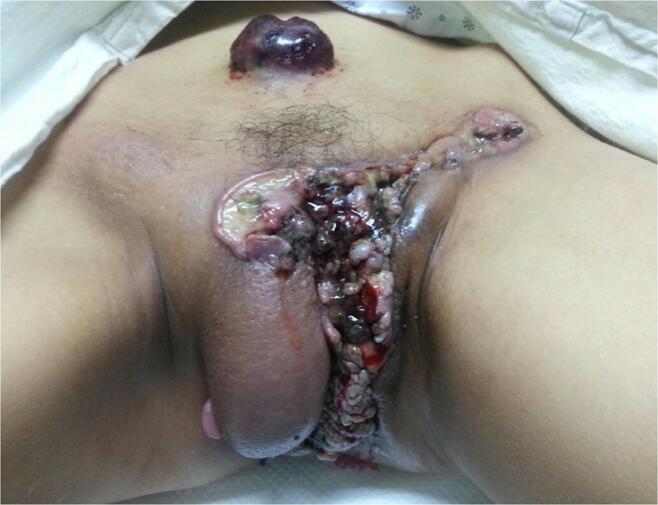
Gross photo shows pubic lesion at initial diagnosis.

In July 2013, a 24-year-old Korean woman referred to the Department of Plastic Surgery of Dong San Medical Center, Daegu, Korea because of a palpable mass in her lower abdomen. Due to the abdominal wall mass and a history of vaginal bleeding since October 2012, the patient was referred to the Department of Obstetrics and Gynecology of Dong San Medical Center. The patient was a nulligravida and had no history of illnesses and no personal or family history of malignancies. A detailed physical examination revealed protruding mushroom-like masses in multiple areas, including her lower abdomen, whole vulva, anus, and both inguinal lesions. The primary lesion comprised a large area of necrotic tissue with exposed bone in the entire vulvar region (Fig. 1). Punch biopsies of the vulva and abdominal skin revealed a malignant tumor consistent with a proximal-type ES. Five days after the first pelvic examination, several bean-sized erythematous nodules on the patient’s scalp and a hyperpigmented bean-sized nodule on her back were detected. Punch biopsies of the scalp and back revealed a malignant tumor suggestive of a metastatic ES.

**Fig. 2:**
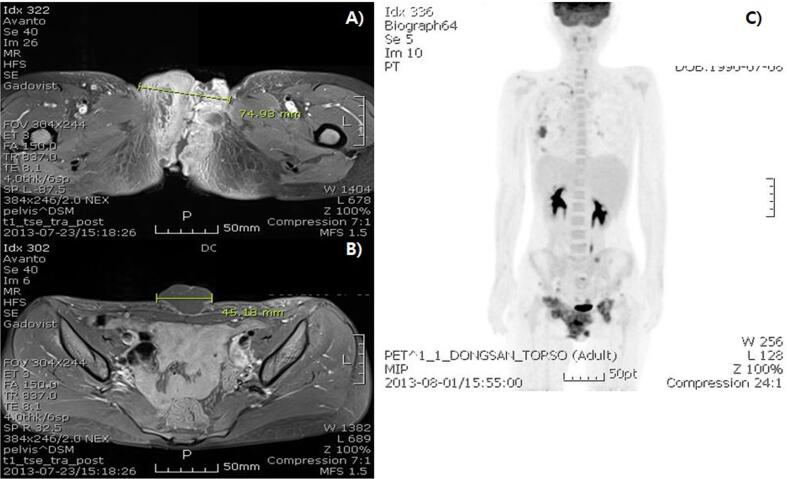
(a) MRI shows large, irregular shaped, infiltrating appearing mass with multifocal intratumoral necrosis at both sides of vulva and perineal area, mainly located at subcutaneous layer. (b) MRI also shows subcutaneous enhancing nodules at lower anterior abdominal wall. (c) PET CT shows distant metastases to lung, left pleura, bone and soft tissue and nodal metastases at left external iliac and right inguinal lesion.

Magnetic resonance imaging of the pelvis showed a large, irregularly shaped infiltrating mass with multifocal intratumoral necrosis on both sides of the vulvar and perineal areas, mainly located in the subcutaneous layer. The size of the main mass was at least 12 × 7 × 8 cm. The mass extended to the lower vagina and the left pelvic wall near the obturator internus area (Fig. 2a). Multiple lymph nodes involving both the common femoral and left external iliac chains and the lower anterior abdominal wall were detected, as well as bone metastasis, including the lower lumbar spine, sacrum, hip bones, and femur (Fig. 2b). Chest computerized tomography (CT) revealed nodules of various sizes in both lungs and pleura at both hemithroax suggestive of metastasis. Positron emission tomography CT showed a sarcoma on both sides of the vulva with intense hypermetabolism invading the perineum, lower vagina, and left inguinal region, as well as nodal metastases in the left external iliac and right inguinal region and distant metastases in the lungs, pleura of the left lung, bones, and soft tissue (Fig. 2c).

**Fig. 3:**
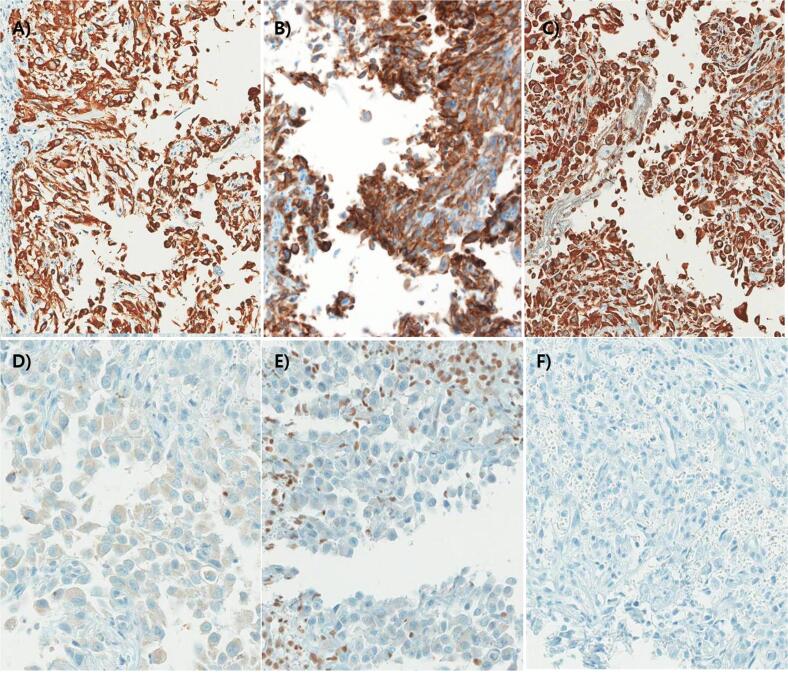
Immunohistochemistry (IHC) of tumor cells reveals diffuse strong expression of cytokeratin (A, x200), epithelial membrane antigen (EMA) (B, x200) and vimentin (C, x200). Additionally, IHC of desmin (D, x400), and myo-D1 (E, x400) shows negative expression in tumor cells. Rhabdoid tumor cells shows loss of INI1 (F, x400).

According to the histological features, the differential diagnoses were malignant melanoma, epithelioid leiomyosarcoma, angiomatoid tumor, rhabdomyosarcoma, metastatic carcinoma, and proximal-type ES. The immunohistochemistry (IHC) results were diffuse and strong positive for epithelial markers of cytokeratin (Fig. 3a), epithelial membrane antigen (Fig. 3b), and mesenchymal marker of vimentin (Fig. 3c). However, IHC of S-100 showed complete negativity for malignant melanomas, desmin for leiomyosarcomas (Fig. 3d), and myo-D1 for rhabdomyosarcomas (Fig. 3e). Moreover, IHC of INI1 revealed a complete loss of tumor nuclei (Fig. 3f), which was indicative of a proximal-type ES.

**Fig. 4:**
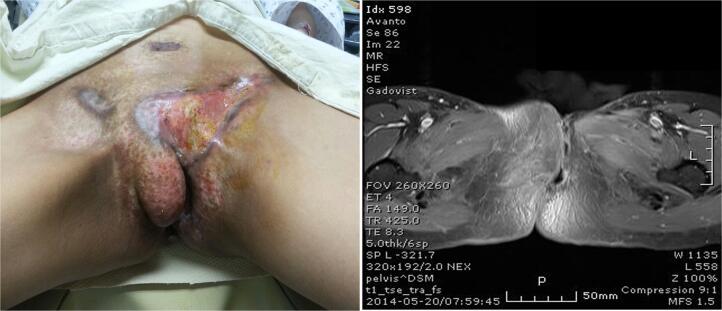
(a) Gross photos show significantly decreased vulvar lesion. (b) MRI shows partial resolution of vulvar lesion.

**Fig. 5:**
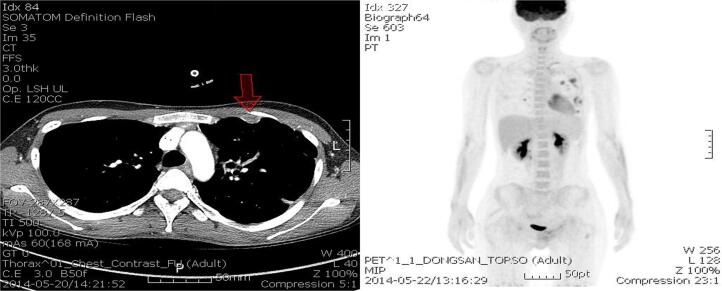
(a) HRCT shows various sized multiple nodules at lungs, fissure, and pleura at both hemithorax. (b) PET CT shows partial resolution of vulvar lesion, complete resolution of pelvic bone metastases and aggravation of multiple lung and left pleura.

The patient’s treatment started with palliative radiation therapy (5,000 cGy/10 fr) of the vulva and abdominal mass for two weeks, followed by two cycles of adjuvant chemotherapy consisting of ifosfamide (1,200 mg/m^2^ on Days 1 to 3). Follow-up imaging studies revealed a good response, with partial resolution of the vulvar lesions and complete resolution of pelvic bone metastasis (Fig. 4). However, one month after treatment cessation, an increased number of lung and pleural lesions were detected, indicating aggravation of distant metastases (Fig. 5). Consequently, the patient received targeted therapy with pazopanib. After two months of a daily regimen of 400 mg of pazopanib, follow-up imaging studies showed partial resolution of the lung and pleural metastases. However, even with an additional three cycles of chemotherapy consisting of cyclophosphamide (500 mg/m^2^), vincristine (1.5 mg/m^2^), and dacarbazine (250 mg/m^2^) on Days 1 to 5, new metastatic lesions were detected in the brain, left mediastinum, neck, and base of the skull. Consequently, radiotherapy was initiated. Nineteen months after the initial diagnosis, the patient expired due to cancer progression and pneumothorax.

## 3 Discussion

To our knowledge, only 39 cases of vulvar ESs have been reported to date (Han et al., 2016, Sundaram et al., 2018, Yue et al., 2018). The low incidence may be due to their rarity and their consequent frequent misdiagnoses as benign lesions. These misdiagnoses often lead to inadequate management or treatment delays. Differential diagnoses include Bartholin’s cysts, benign inflammatory lesions, necrotizing granulomas or other sarcomas with epithelioid cells, malignant rhabdoid tumors, melanomas (including sarcomatoid melanomas), and undifferentiated carcinomas (Iavazzo et al., 2014). ESs often occur in the labia majora of young women, with the most common initial symptom being a slowly growing, relatively painless mass.

Most ESs in the upper and lower extremities of young adults are of the distal type, whereas ESs in the gastrointestinal tract, urogenital organs, and vulva are of the proximal type (Kim et al., 2008). Han et al. (Han et al., 2016) reported a mean ES patient age of 38.6 years, ranging from 17 to 84 years. They estimated the overall peak incidence of ESs at around 35 years of age, and most patients in previous reports were aged 35 years or younger. Irrespective of the patient’s age at the time of the initial diagnosis, no previous studies have reported distant metastasis in an ES patient at the time of the initial diagnosis.

Microscopically, there was a complete loss of tumor nuclei in IHC of INI1 of this case. Biallelic inactivation (homozygous deletion or mutation/deletion) of INI1 (also known as hSNF5 or SMARCB1) is observed in malignant rhabdoid tumors in infancy and in atypical teratoid/rhabdoid tumors (Han et al., 2016). Previous research has established the utility of immunohistochemical staining for the INI1 protein in confirming the diagnosis of malignant rhabdoid tumors and atypical teratoid/rhabdoid tumors by demonstrating a loss of expression. Using IHC, recent studies have also reported that the loss of INI1 expression is associated with ESs, especially of the proximal type (Roberts and Biegel, 2009).

In a previous study that included 37 ES patients, the initial treatment in all cases was a surgical approach, ranging from local excision to radical vulvectomy, with or without node dissection. The latter was possible due to the absence of distant metastasis. Many studies have reported wide local excisions, with surgical margins of at least 2 cm, rather than radical vulvectomies (Roberts and Biegel, 2009). However, the optimal treatment remains uncertain. In a previous study of 37 ES patients, 14 patients received adjuvant therapy, including radiation therapy (10/37, 27%) and chemotherapy (7/37, 18.9%), due to recurrence. Among 31 patients of proximal-type vulvar ESs, Iavazzo et al. (Iavazzo et al., 2014) reported a recurrence rate of 42%, with the lungs and lymph nodes being the most common sites of recurrence.

The role of adjuvant radiotherapy and/or chemotherapy in ES treatment remains unclear. Due to the poor prognosis owing to the aggressive nature of ESs, there are currently no accepted standardized treatment guidelines. A previous study found that despite palliative chemotherapy, the prognosis of ES patients with metastasis was poor, with one- and five-year survival rates of 42% and 0%, respectively (Rego et al., 2015).

Radiotherapy is known to be less effective against sarcomas. However, in our case, radiotherapy was the most effective treatment in reducing the size of the main lesion in the vulva. Combination chemotherapy including ifosfamide elicited a partial response. Ifosfamide and anthracyclines are known the only active agents in advanced soft tissue sarcomas (Jones et al., 2012). In this case only single regimen of ifosfamide was administered along with the radiotherapy in the concept of concurrent chemoradiotherapy. Targeted therapy with pazopanib, an inhibitor of the vascular endothelial growth factor receptor pathway and the first non-chemotherapeutic anticancer agent approved by regulatory authorities for soft tissue sarcomas, also resulted in a partial response (Van Der Graaf et al., 2012). Given the absence of treatment options for ESs, pazopanib was considered a possible option at the time. However, although pazopanib may be used as the first targeted therapy in advanced ES, it has not achieved satisfactory results to date (Frezza et al., 2018). Systemic therapy for ES is not supported by conclusive evidence or a substantial number of cases. Based on research into previous generations of treatments, anthracycline-based regimens (anthracycline alone or combined with ifosfamide) were previously regarded as first-line treatments in advanced or metastatic ES. After the U.S. Food and Drug Administration approved tazemetostat, an oral inhibitor of EZH2, for the treatment of patients aged 16 years or older with metastatic or advanced ESs that cannot be radically resected, the trend has been changing. Tazemetostat has been shown to be safe and effective in clinical trials and is now the most actively used alternative to anthracycline (Czarnecka et al., 2020). A phase Ib/III trial of tazemetostat combined with doxorubicin as a first-line therapy for advanced ES is currently underway (Sen et al., 2020).

In our case, the patient died following disease progression. Palliative radiotherapy followed by chemotherapy resulted in an overall survival of 19 months. Few cases of advanced vulvar ESs with metastases to other organs at the time of the initial diagnosis have been reported. Due to aggressive disease progression without proper treatment, cases of sudden death within a day of the diagnosis have been reported (Rodrigues et al., 2015, Yee et al., 2016).

Most cases of vulvar ESs are detected at an early stage, without regional and/or distant metastasis, and may or may not require adjuvant therapy. Our case is interesting because it is the first case of a vulvar ES not only with multiple distant metastases but also with an extremely large lesion at the time of the initial diagnosis. This case also demonstrates the use of possible treatment options, including radiotherapy and target therapy, in an ES with distant metastasis. Patients with metastatic or advanced ESs may benefit from multimodal approaches. Depending on the number and location of metastatic lesions, it is necessary to consider various treatment approaches. In selected patients, not only surgery and systemic chemotherapy but also radiotherapy and/or target therapy should be considered to achieve optimal results and prolong overall survival.

## Declaration of Competing Interest

The authors declare that they have no known competing financial interests or personal relationships that could have appeared to influence the work reported in this paper.
